# Suicide Research, Prevention, and COVID-19: Towards a Global Response and the Establishment of an International Research Collaboration

**DOI:** 10.1027/0227-5910/a000731

**Published:** 2020-07-27

**Authors:** Thomas Niederkrotenthaler, David Gunnell, Ella Arensman, Jane Pirkis, Louis Appleby, Keith Hawton, Ann John, Nav Kapur, Murad Khan, Rory C. O'Connor, Steve Platt, The International COVID-19 Suicide Prevention Research Collaboration

**Affiliations:** ^1^ Unit Suicide Research and Mental Health Promotion, Department of Social and Preventive Medicine, Centre for Public Health, Medical University of Vienna, Austria; ^2^ National Institute of Health Research Biomedical Research Centre, University Hospitals Bristol NHS Foundation Trust and the University of Bristol, UK; ^3^ School of Public Health and National Suicide Research Foundation, College of Medicine and Health, University College Cork, Republic of Ireland; ^4^ Melbourne School of Population and Global Health, University of Melbourne, VIC, Australia; ^5^ Centre for Mental Health & Safety, The University of Manchester, UK; ^6^ Centre for Suicide Research, Department of Psychiatry, Warneford Hospital, University of Oxford, UK; ^7^ Population Psychiatry, Suicide and Informatics, Medical School, Swansea University, UK; ^8^ Centre for Mental Health and Safety & Greater Manchester NIHR Patient Safety Translational Research Centre, University of Manchester and Greater Manchester Mental Health NHS Foundation Trust, Manchester, UK; ^9^ Department of Psychiatry, Aga Khan University, Karachi, Pakistan; ^10^ Suicidal Behaviour Research Laboratory, Institute of Health & Wellbeing, University of Glasgow, UK; ^11^ Usher Institute, College of Medicine and Veterinary Medicine, University of Edinburgh, UK; ^12^ Pablo Analuisa, Louis Appleby, Ella Arensman, Jose Luis Ayuso-Mateos, Jason Bantjes, Jose Bertolote, Eric Caine, Lai Fong Chan, Shu-Sen Chang, Ying-Yeh Chen, Helen Christensen, Rakhi Dandona, Diego De Leo, Michael Eddleston, Annette Erlangsen, David Gunnell, Jill Harkavy-Friedman, Keith Hawton, Ann John, Fabrice Jollant, Nav Kapur, Murad Khan, Olivia J. Kirtley, Duleeka Knipe, Kairi Kolves, Flemming Konradsen, Shiwei Liu, Sally McManus, Lars Mehlum, Matt Miller, Ellenor Mittendorfer-Rutz, Paul Moran, Jacqui Morrissey, Christine Moutier, Thomas Niederkrotenthaler, Emma Nielsen, Merete Nordentoft, Rory O’Connor, Siobhan O’Neill, Maria Oquendo, Joseph Osafo, Andrew Page, Michael R. Phillips, Jane Pirkis, Steve Platt, Boris Polozhy, Maurizio Pompili, Ping Qin, Thilini Rajapakse, Mohsen Rezaeian, Barbara Schneider, Morton M. Silverman, Mark Sinyor, Steven Stack, Ellen Townsend, Gustavo Turecki, Michiko Ueda, Lakshmi Vijayakumar, Paul Yip, Gil Zalsman

The COVID-19 pandemic of 2020 is a major global health challenge. At the time of writing, over 11.6 million people around the world had been registered as infected and 538,000 had died (Worldometers, 2020, accessed July 7, 2020). Public health responses to COVID-19 need to balance direct efforts to control the disease and its impact on health systems, infected people, and their families with the impacts from associated mitigating interventions. Such impacts include social isolation, school closure, health service disruption stemming from reconfiguring health systems, and diminished economic activity. The primary focus of both the United Nations (UN) and the World Health Organization (WHO) has been on addressing COVID-19 as a physical health crisis, but the need to strengthen mental health action, including suicide prevention, is increasingly recognized, as is the need for mental health research to be an integral part of the recovery plan (UN, 2020a). The impacts of the pandemic on physical and mental health will unfold differently over time and will vary depending on the duration and fluctuating intensity of the disease. Research is needed to help ensure that decision-making regarding all aspects of health, including mental health (Holmes et al., 2020), is informed by the best quality data at each stage of the pandemic.

The pandemic poses a prolonged and unique challenge to public mental health, with major implications for suicide and suicide prevention (Gunnell et al., 2020; Reger, Stanley, & Joiner, 2020). A rise in suicide deaths in the wake of the pandemic is not inevitable. There is consensus, however, that the mitigation of risk will be contingent upon a proactive and effective response involving collaborative work between the state, NGOs, academia, and local governments and coordinated leadership across government ministries, including health, education, security, social services, welfare, and finance. Countries have responded in different ways to the pandemic, effectively creating a series of natural experiments. Thus, regions of the world affected later in the pandemic can draw on lessons from countries, such as China and Italy, affected in its early phase. Likewise, lessons learned early in the pandemic (e.g., on the impact of lockdown and physical distancing measures) can be used to inform responses to any future surges in the incidence of COVID-19.

Although there are important parallels between countries in the course of the pandemic, some stressors, responses, and priorities are likely to differ between high- and low–middle-income countries and between cultures and regions. As COVID-19 appears to be disproportionately affecting Black, Asian, and minority ethnic communities, the response – and suicide prevention research carried out to inform the response – needs to be sufficiently granular and account for the complexity of risks in these groups (O'Connor et al., 2020).

Throughout this editorial, when we refer to *suicide* and *suicidal behavior*, we mean to include both fatal and nonfatal suicidal behaviors and self-harm.

## The Need for Evidence-Based Suicide Prevention Responses 

Suicide is the most extreme outcome of a mental health crisis and should therefore be a key priority in any broader mental health response to the pandemic (Gunnell et al., 2020; Reger et al., 2020). Suicide prevention responses need to be informed by research that is as specific as possible to the current situation and takes account of the many mechanisms that have an impact on suicide, as they may vary during the different phases of the pandemic. At the same time, given the risks involved, strategic development of policy and implementation responses cannot wait until all aspects of the epidemiology and consequences of the disease on mental health and risk of suicide are understood. 

The dilemma here is that few studies have investigated the impact of previous pandemics – or even epidemics – on suicide (Cheung, Chau, & Yip, 2008; Wasserman, 1992; Zortea et al., 2020), and none has evaluated suicide prevention measures in the current context. An analysis of the impact of the Spanish Flu epidemic (1918–1920) in the United States indicated that it resulted in a small rise in suicides (Wasserman, 1992). Cheung and colleagues (2008) reported a rise in suicide among older people during the 2003 SARS epidemic in Hong Kong. Similarly, what can be learned from other types of public health emergencies is limited. Much of the related research comes from one-off events, such as terrorist attacks and natural disasters (e.g., earthquakes). Findings from such events might not be applicable to the current situation (Devitt, 2020).

## Early Research Findings Relevant to Assessing the Impact of COVID-19 on Mental Health 

Early publications relevant to the COVID-19 response have largely come from literature reviews, small selective surveys or case reports, often using indirect measures of suicide risk or from modeling approaches to predict the impact of the pandemic. These have addressed issues such as the impact of quarantine (Brooks et al, 2020), highlighted possible high-risk groups (Yao, Chen, & Xu, 2020), and assessed mental health service disruption (Royal College of Psychiatrists, 2020). 

Physical distancing and related measures, which have been at the forefront of the public health response, carry a strong risk of increasing isolation, particularly in vulnerable populations such as older people and people who have been bereaved (Brooks et al., 2020; De Leo & Trabucchi, 2020; Wand, Zhong, Chiu, Draper, & De Leo, 2020; Yip & Chau, 2020). Physical distancing measures may also lead to increases in household stress levels, domestic violence, and alcohol misuse and affect the accessibility of mental health services (Brooks et al., 2020; Reger et al., 2020). The stresses of lockdown may be worse in low- and middle-income countries where extended families tend to live together with limited housing space. 

Concerns have been expressed about increases in demand for psychiatric emergency care (Royal College of Psychiatrists, 2020). In the context of overwhelmed health-care systems and shortages of resources to treat people with COVID-19 in healthcare settings, qualitative findings from China indicate that the intensity of work during the pandemic drained health-care workers physically and emotionally (Liu et al., 2020). In the United Kingdom, the British Medical Association well-being support services have seen a 40% increase in use after the onset of the pandemic (Torjesen, 2020). 

Positive effects of the pandemic on the public, such as increased prosocial behavior (e.g., donating and volunteering) and the strengthening of community ties, may help to mitigate detrimental impacts of physical distancing (Van Bavel et al., 2020). The move of some health and third-sector services into online settings may also have long-lasting benefits in improving service accessibility, particularly to those who find face-to-face consultation difficult. The effect on people with mental illness of replacing face-to-face treatment with remote delivery of care, however, remains unclear. Moreover, in low- and middle-income countries the technology to support remote assessment is limited (De Sousa, Mohandas, & Javed, 2020; UN, 2020a). In these, and other settings, where there is limited access to specialist mental health services, community and peer support becomes extremely important. 

The potential for the COVID-19 virus to affect the brain and to cause long-lasting physical morbidity means it might become relevant as a risk factor for mental illness and suicide in the future (Holmes et al., 2020; Rogers et al., 2020; Wu et al. 2020). Review findings indicate that the incidence of psychosis, a major risk factor for suicide and suicidal behavior, appeared to be high in people following SARS, MERS, and H1N1 infection (Rogers et al., 2020). Given emerging evidence that the virus can have severe effects on different organ systems including kidney and liver function (Zhang, Shi, & Wang, 2020), the physical consequences of infections might include a prolonged reduction in functional capacity and disability in some patients, all of which might have potential implications for suicide risk and prevention. 

However, longer-term risks for suicide are likely most closely related to the economic consequences of the pandemic, including financial strain and unemployment. In a study based on suicide data from 54 countries, the recession of 2008 was associated with a 3.3% increase in suicides in men (but not women) in the following year and more prolonged increases in several countries (Chang, Stuckler, Yip, & Gunnell, 2013). The increase varied depending on the regional depth of the recession and the specifics of the social insurance systems (e.g., regulations for unemployment benefits or payed sick leave; Chang et al., 2013; Norström & Grönqvist, 2015). The economic downturn associated with the COVID-19 pandemic may be more rapid in onset than the 2008 recession and may push an estimated 500 million people, particularly in low- and middle-income countries, below the poverty line (UN, 2020b). 

## Early Research Findings Relevant to Assessing the Impact of COVID-19 on Suicide and Suicidal Behavior 

There is, as yet, no direct evidence of the impact of the pandemic on suicidal behavior. While a number of news stories from Japan, New Zealand, and Germany report a decrease in suicides in the period around the time of lockdown (Deutsche Welle, 2020; New Zealand Herald, 2020; The Guardian, 2020), these are all based on preliminary data/anecdotal reports and unsubstantiated by peer-reviewed publications. General population survey findings from the United Kingdom have shown no clear evidence of a rise in reported self-harm during the weeks following lockdown (after March 23), but no pre-lockdown data are available (Fancourt, Bu, Mak, & Steptoe, 2020). Many surveys have been carried out in the wake of the pandemic, these often use convenience samples, which are prone to selection bias (Pierce et al., 2020). In addition, there have been multiple case reports from some low- and middle-income countries highlighting occurrences of suicide thought to be related to COVID-19 (De Sousa et al., 2020; Mamun & Ullah, 2020). These reports must, however, be interpreted with great caution – and even more so when they are based on mass media reports, which are unlikely to have been validated. 

Some researchers have attempted to model the possible pandemic-associated increase in suicides, largely based on predicted rises in unemployment (Kawohl & Nordt, 2020; McIntyre & Lee, 2020; Moser, Glaus, Frangou, & Schechter, 2020). Risk estimates vary widely, from a 1% increase in global suicides (Kawohl & Nordt, 2020) to a doubling of national suicides in a Swiss study, using prison incarceration as a questionable proxy for modeling the social distancing effects of lockdown (Moser et al., 2020). These discrepancies are partly due to differences in modeling assumptions, which are associated with considerable uncertainty and may be very misleading. Given the uncertainty of the baseline assumptions about how events will unfold, the results of these tentative projections can at best provide a guide as to where action should be directed but are largely unhelpful for accurate quantifications of future suicidal behavior and suicide.

 In this regard, access to real-time suicide mortality data is a key priority (Gunnell et al., 2020). Further, active surveillance systems for suicide attempts are warranted (WHO, 2016).

In the absence of direct evidence about trends in suicide, some researchers have used search behavior on Google Trends for terms related to suicide, as a proxy for suicide risk (Knipe, Evans, Marchant, Gunnell, & John, 2020; Sinyor, Spittal, & Niederkrotenthaler, 2020). Their findings indicate that, although relative search volumes for financial and work-related concerns have increased (Knipe et al., 2020), searches for suicide and suicide methods have not (Knipe et al, 2020; Sinyor et al., 2020). The potential limitations of Google search data for surveillance are well recognized and include uncertainty about the algorithms used and issues with the stability of findings provided by Google Trends, as well of inconsistent associations with suicide (Tran et al., 2017).

Gaps in knowledge about the epidemiology of suicide and suicidal behavior during COVID-19 and the effectiveness of intervention and prevention measures underline the need for a strategic approach to suicide research and prevention at a global level. The uncertainties regarding the direct and indirect effects of COVID-19 on suicide can only be addressed with good-quality tailored research. Furthermore, suicide prevention in the age of COVID-19 needs to build on what we know about the effectiveness of various measures, but also needs to take account of the unique challenges posed by the situation in order to develop novel approaches. Our knowledge is currently still very limited and building the evidence base on suicide prevention is crucial. 

## Research Considerations During COVID-19 

There are several considerations in relation to suicide prevention research carried out during crisis situations and in the present global pandemic (Table 1). These include ensuring the safety of research participants and researchers as well as the need for research to focus on low- and middle-income settings as well as high-income countries, keeping in mind that findings from one setting may not generalize to another. We expand on a few specific issues in the following section. First, the limited research conducted thus far on suicide and its prevention during COVID-19 has focused mostly on high-income countries. While complementary research in this area in low- and middle-income countries should be prioritized, the poor quality of routine mortality and hospital attendance data as well as the limited availability of resources to carry out research in many of these settings present very real challenges. In 2014 the WHO considered that only just over one third of member states had good-quality suicide registration data, and such data were largely absent in low- and middle-income countries (WHO, 2014). The establishment of sentinel sites to gather as accurate data on suicidal behavior as possible to supplement those that already exist would be one way forward (WHO, 2016). 

**Table 1:** Considerations for suicide and suicidal behavior research during the COVID-19 pandemic

Research considerations
The COVID-19 suicide research response should be truly multidisciplinary. This will foster research that addresses the different aspects and layers of risk and resilience relating to the health consequences of COVID-19, including suicide and suicidal behavior. It will also foster research that informs prevention efforts by taking a range of perspectives.
People with lived experience of suicide should be involved at all stages of the research process.
Researchers should ensure that key risk groups that are often under-represented in suicide research are represented appropriately in studies.
The safety and well-being of participants should remain at the forefront of research design considerations.
Researchers' safety must not be compromised if they are carrying out field work in situations where they may be at increased risk of infection.
Researchers should embrace Open Science research practices, such as registering research questions in advance and sharing data, wherever possible.
To ensure research findings inform practice, researchers should consider the potential real-world impact of their studies during the design phase and develop a clear, a priori dissemination strategy.
Research findings, particularly those making bold statements about risk or about effective treatments, should be peer reviewed prior to dissemination. If researchers decide that early dissemination is warranted, outputs should clearly state the preliminary status of the research and that it is yet to be peer reviewed. In this case, conclusions should be stated cautiously, in a manner that is consistent with the preliminary nature of findings.
When talking about research findings with the media, researchers should remain vigilant about not increasing risk for people who are already vulnerable. They should take care not to contribute to sensationalist headlines, should not make monocausal attributions of suicide to COVID-19, and should not use stigmatizing language (e.g., COVID-19 suicides). Researchers should recommend that media professionals use COVID-19-specific media reporting guidelines (see IASP, 2020b).
Research teams should be supported, particularly because some team members will be working in difficult home circumstances and many will be personally affected by concerns about the pandemic and its consequences.

Second, as a result of the pandemic, mental health services have had to develop new ways of working to deliver care to suicidal individuals, including new care pathways, the mass roll-out of remote consultation, and increased use of digital interventions. These new ways of working require real-time evaluation and ongoing adaptation in response to findings. Traditional evaluation approaches, such as randomized trials, may need to be adapted in a manner that is still consistent with making robust inferences about their effectiveness.

Third, with school and university closures in place in a number of countries, the traditional setting for carrying out research into children and young people's health is no longer available. Given current concerns about the impact of the pandemic on young people, mental health researchers will need to find alternative routes to studying the impact of the pandemic on this potentially vulnerable group. 

Fourth, for all studies it is vital that those with lived experience of suicide are involved in shaping the research at all stages – from developing the research questions to data collection and dissemination of the findings. Fifth, all research needs to comply with ethical standards. Researchers who do not normally work in the area of mental health and suicide prevention but who are now shaping conversations on suicide prevention need to obtain necessary training and background information on how to conduct suicide research, including the need to follow established research protocols and safety considerations that are specific to the field (Townsend, Nielsen, Allister, & Cassidy, 2020). Sixth, it is important that research resources (i.e., staff, funding) are rapidly mobilized to ensure timely research evidence is available. However, this presents tensions between the time researchers have available to write robust funding applications, time-scales for the grant review by funding bodies, and, if funded, the availability of high-quality fieldworkers and analysts as these are likely to be already committed to other projects. Flexibility and clear communication with funders about project delays and re-allocation of resources should help ameliorate these challenges. There is a distinct possibility that research funding may be adversely affected by a post-pandemic recession. Seventh, any proposed research should have a clear pathway to impact to ensure that clinicians and policy-makers can implement the findings of research in their work.

Lastly, traditional models of research publication, with the need for peer review, introduce delays between article submission and on-line publication, reducing the speed with which evidence is disseminated and recommendations implemented. One solution is the fast-track review processes for selected papers – these were already in place before COVID-19, but have been extended and adopted by more journals since the beginning of the outbreak. Another solution is open science publication models that involve on-line publication of articles while they await peer review, although there is a danger of low-quality research findings being disseminated and acted upon precipitously, without scrutiny of their validity (Armstrong, 2020). In order to mitigate this risk, researchers need to label their findings as preliminary and implement a communications strategy that addresses the preliminary nature of findings. 

## The International COVID-19 Suicide Prevention Research Collaboration 

High-quality timely research to understand the suicide-related consequences of COVID-19 and to determine how best to mitigate the risk stemming from these consequences is now needed. The UN highlights the need for "rapid knowledge acquisition," establishing research priorities, coordinating research efforts, open-data sharing, and funding (UN, 2020a). In response to widespread concerns about the impact of the COVID-19 pandemic on suicide and suicidal behavior, a group, initially consisting of 44 suicide prevention researchers and leaders of suicide prevention charities from around 20 countries, came together to pool their expertise about the likely impact of the pandemic on suicidal behavior and to identify prevention priorities. The International COVID-19 Suicide Prevention Research Collaboration (ICSPRC) sought to include at least one representative from many of the most affected countries and also representation from high-, middle-, and low-income countries (https://www.iasp.info/COVID-19_suicide_research.php). The ICSPRC assessment of the risks posed by the pandemic and suggested responses to mitigate these were summarized in a *Lancet Psychiatry* commentary published in April 2020 (Gunnell et al., 2020). 

Building on this initiative, the collaborative network has been extended to include suicide researchers from a wider range of countries (including countries in Africa, the Middle East, and South America), with skills ranging from population health to biological psychiatry and incorporating expertise in quantitative and qualitative methods, together with ethics. The objectives of the group are to:

a) Share early findings (and, where appropriate, data) on the impact of the pandemic, and the public health measures (e.g., physical distancing) to contain its spread, on suicidal behavior in participants' countries and to provide timely policy advice to those in other countries.
b) Facilitate collaboration/avoid duplication through sharing information about ongoing research studies and COVID-19 research tools/questionnaires focused on suicide prevention, as well as advice about study design.
c) Harmonize data collection approaches to facilitate pooling of data, where possible, from different settings and contexts.

An early example of the success of this approach has been the collaboration between two groups working on almost identical systematic reviews investigating the impact of pandemics/epidemics on suicide, self-harm, and suicidal ideation (Zortea et al., 2020). Another group has established real-time surveillance of the emerging literature on COVID-19 and suicide to become a "living review" (John et al., 2020). The global distribution of group members will facilitate rapid combined efforts in response to funding opportunities, where cross-national studies would strengthen the evidence base. 

Conducting high quality suicide prevention research is challenging. Suicide, in population terms, is a low-incidence event and thus studies are often under-powered to identify small but potentially important effects. Furthermore, a focus on intermediate or proxy outcomes (e.g., self-reported suicidal ideation) is sometimes necessary but these have a questionable relationship to suicidal behaviors (Mars et al., 2019; May & Klonsky, 2016). The collaboration provides a mechanism to work together, pool data using shared protocols, and investigate different outcomes with a range of research designs. It should also facilitate reaching global consensus on issues such as the impact of lockdown on suicide risk and how best to mitigate risk, especially if further periods are required to address the re-emergence of COVID-19, as has recently been reported in countries such as Iran (Worldometers, 2020). 

The collaboration has identified several suggestions for research to help inform responses to the current and future pandemics, formulating these as research questions (see Table 2 and Table 3). The proposed research questions link to the gaps in knowledge that we identified earlier. Table 2 highlights research questions relating to whether rates of suicidal behavior increase as a result of the pandemic and what mechanisms may be driving any increase, suggesting specific research for the general population and for high-risk groups. Table 3 presents research questions relating to whether particular responses might help to mitigate any risk of suicide associated with the pandemic. Members of the collaboration have worked with the International Association for Suicide Prevention (IASP) to establish a searchable on-line list of ongoing COVID-relevant studies on suicidal behavior, managing suicidal crises, and suicide prevention (https://www.iasp.info/covid-19/covid-19-sui​cide-research-studies) to facilitate collaboration and avoid duplication, similar to the website developed for longitudinal studies on mental health during COVID-19 (https://www.covidminds.org/longitudinalstudies). The role of the IASP, in collaboration with other international and national organizations (e.g., WHO, International Association of Suicide Research [IASR], American Foundation for Suicide Prevention [AFSP], and others), is to provide up-to-date information on suicide research and prevention in its global network. The IASP is developing a strategic plan to reduce COVID-19-related suicidal behavior and building a central pool of resources (expertise, research, guidelines for good practice, briefings) that will be available to support organizations globally (IASP, 2020a, b). Members of the ICSPRC have contributed to an IASP briefing paper on reporting suicide during the COVID-19 pandemic and IASP members have developed guidance to help workplaces and professional associations through the COVID-19 Crisis (IASP, 2020a, b). The combination of the specific research focus in the ICSPRC and IASP, with its prevention network and links to the WHO, as the leading organization for suicide prevention globally, is a core strength of this collaboration and many members are active in both. 

**Table 2:** Example research questions relating to whether rates of suicide and/or suicidal behavior increase as a result of the pandemic and what mechanisms may be driving any increase

**General population**
Variations across time, place, and person
Time	What is the impact of the pandemic on suicide and suicidal behavior and does risk differ over its course and in its aftermath?
Place	Are there underlying country- or region-level differences that may explain differing changes in rates of suicide and suicidal behavior? For example, do the background rates of suicide and suicidal behavior seem to have a bearing on any increases? What about the number of COVID-19 cases and deaths, the capacity of the health-care system, and the pandemic response? Are any observed relationships the same for low- and middle-income countries as they are for high-income countries?
Person	Does any change in the incidence of suicide and suicidal behavior vary by population subgroup? For example, is there variation by demographic factors (e.g., age, gender, ethnicity, religious affiliation), household structure (e.g., living alone, living with children, living with joint/extended families), socioeconomic factors (e.g., socioeconomic status, job loss, financial strain, debt, access to resources, occupation)?
Risk, protective, and new individual-level factors
Risk factors	Are there recognized risk factors for suicide and suicidal behavior that are heightened during the pandemic that might explain any increases? For example, how do any changes in suicide and suicidal behavior relate to changes in levels of anxiety, depression, alcohol use, or feelings of entrapment that might be increased by isolation, loneliness, uncertainty, domestic violence, economic hardship, and reduced social participation?
Protective factors	Are there recognized protective factors for suicide and suicidal behavior that might be bolstered during the pandemic and potentially keep rates of suicide and suicidal behavior from increasing? For example, if communities rally around and provide support for those who might be vulnerable, does this have a positive impact?
New factors	Are there new risk or protective factors for suicide and suicidal behavior that correspond to the emergence of the pandemic? Are there risk or protective factors that have been exacerbated or changed in importance? For example, has face-to-face and online racism against Asian people during the pandemic led to an increase in their risk of suicidal behavior?
Population-level factors
Access to the means	Have changes in access to the means of suicide resulted in changes to methods used and affected rates of suicide and suicidal behavior? For example, has suicide by firearms, pesticides, and medications increased as a result of people stockpiling these? Have rail suicides decreased due to travel restrictions?
Media reporting	How does the media report on COVID-19 and on COVID-19-related suicides, and what is the impact of this reporting on suicide and suicidal behavior? For example, are suicide-related narratives different compared with pre-COVID-19?
Social media use and other online activity	Have patterns of social media usage/consumption and other online activity changed during the pandemic, and, if so, is this associated with suicidal behavior? For example, does repeated exposure to information about the pandemic heighten fear and increase the risk of suicide and suicidal behavior? Or does the connectedness afforded by digital technologies counter the isolation effects of physical distancing?
Ways we live and behave	Has the pandemic changed the way we live and behave, or will it do so in the future? If so, which changes are beneficial and which are harmful with respect to the risk of suicide and suicidal behavior?
**High-risk groups**
COVID-19 related high-risk groups
Bereaved	Are people who have lost someone to COVID-19 at increased risk of suicide and suicidal behavior?
Vulnerability to COVID-19	Do people who may be particularly vulnerable to COVID-19 (e.g., older people, those living with chronic conditions or other medical complications) have elevated risk of suicide and suicidal behavior?
Infected by COVID-19	Are people who are recovering or have recovered from COVID-19 at increased risk of suicidal behavior? Are there neurobiological mechanisms that mediate any increased risk for these people? Are people experiencing longer-term physical consequences of COVID-19 infection at increased risk of suicide and suicidal behavior?
Frontline care workers	Is there an increased risk of suicide and suicidal behavior among frontline health and social care staff who are looking after patients withCOVID-19? If so, is this risk associated with exposure to the virus, loss and grief, ethical challenges of having to make unprecedented choices, or something else?
Other high-risk groups
Mental health problems	Has the risk of suicide and suicidal behavior increased for people with pre-existing mental health problems?
Suicide attempters	Are there increased numbers of people who make suicide attempts without presenting to hospital?
Economically affected and high-risk occupational groups	Are people whose economic circumstances have been adversely affected by the pandemic (e.g., those who have lost their jobs, those whose businesses have folded) at increased risk of suicide and suicidal behavior? What is the impact of COVID-19 on suicide and suicidal behavior in different occupational groups, e.g., health-care staff, those working in the retail sector, artists, and groups in precarious working conditions?
Young people	Are children and adolescents at increased risk of suicidal behavior as a result of factors such as changes to their educational and vocational opportunities and reduced face-to-face contact with their peers? Are there particularly sensitive developmental stages or ages where interruptions will have the greatest impact on suicide and suicidal behavior in adulthood? What is the impact of COVID-19 on trends in suicide and suicidal behavior among children, adolescents, and young adults?
Older people	Are older people at increased risk? What are the impacts of bereavement, loneliness, vulnerability to COVID-19, and stigma? Do living arrangements (e.g., living alone, living in aged care facilities) have an influence on risk?
Migrants/refugees and displaced people	Is there an increased risk of suicide and suicidal behavior for migrants who may be living without a job in their host country or being forced to return to their native country? And what about refugees and displaced people living in camps with limited access to support or care? Are there differences between migrant groups and are refugees, asylum-seekers, and irregular migrants at increased risk of suicidal behavior?

**Table 3:** Example research questions relating to whether particular approaches/responses might help to mitigate any risk of suicide and/or suicidal behavior associated with the pandemic

Mental health consequences of lockdown	Are there country- or region-level differences in the pandemic response that are associated with greater or lesser changes in rates of suicide and suicidal behavior? For example, does the timing, scale, intensity, and duration of lockdown make a difference? What about the way physical distancing measures are enforced? Does the extent to which the public buys into and observes the restrictions (and whether this changes as time goes by) impact on any changes seen in suicide and suicidal behavior? Does it make a difference whether the lockdown is a well-articulated and coordinated national strategy or whether it is more fragmented in its conceptualization and implementation?
	Do rates of suicide and suicidal behavior vary across different stages of the pandemic (e.g., during lockdown, once restrictions are eased), and what does this tell us about particular lockdown policies? Similarly, do sales of prescription psychotropic medication and/or use of mental health services vary by pandemic stage, and what can we learn from this?
	How can social networks be activated to identify and provide support to people who may be struggling due to lockdown?
	How can care best be delivered to suicidal individuals when people are unable or afraid to leave their homes?
Economic consequences of the pandemic	What can we learn from responses to previous pandemics or epidemics (e.g., the 2003 SARS outbreak) and economic crises (e.g., the 2008 recession) to inform our response to COVID-19?
	Are there country- or region-level differences in economic responses that are associated with greater or lesser changes in rates of suicide and suicidal behavior? For example, do income guarantees, employment protection, and labor market programs make a difference? What about equity of access to resource provision?
Burden of mortality from COVID-19	How can care best be provided to individuals who have been bereaved through COVID-19 and to individuals who have been bereaved by suicide during the pandemic?
	What sort of interventions might improve media reporting in relation to deaths due to COVID-19 and suicides during the pandemic?
Health-care and crisis line responses	How is availability of mental health services related to risk of suicide and suicidal behavior? Are there ways of scaling up mental health-care delivery?
	How does knowledge of sociodemographic and clinical risk factors and neurobiological mechanisms inform prevention/treatment approaches?
	What are the best ways to reach out to people who are not in touch with services? How can we encourage help-seeking?
	How can mental health services best be delivered to suicidal individuals during the pandemic? How well do telehealth and online options work? Is it possible to identify and evaluate new forms of health-care services based on experiences and adaptations during the pandemic?
	How can general health and mental health professionals be trained to respond effectively to suicidal clients or patients during the course of the pandemic? Do they need to learn new ways of operating?
	Have crisis lines providing telephone and online chat support been used as resources for suicide prevention during the pandemic? Has the use of these sorts of services by suicidal individuals increased? Do people find them helpful?
Workplaces and educational institutions	How can workplaces help mitigate the risk of suicide and suicidal behavior during the pandemic? Are there ways they can support workers who may be working fewer hours or taking home less pay? And can they play a role in helping workers who may have lost their jobs?
	How can schools and universities keep students positive, motivated, and safe during the pandemic?

A key issue the group needs to consider is how best to ensure the rapid dissemination of research and surveillance information to inform policy-making and prevention activities. Furthermore, there is a need to consider the best way of responding to (sometimes unsubstantiated) findings reported in news articles that may be hastily picked up by policy-makers and politicians. Three sorts of information are relevant: (a) routinely available data (e.g., national mortality, survey data, research publications) that not everyone will be aware of – this could be disseminated via regular briefings/updates; (b) pre-publication research data and findings that may inform policy, but are going through peer review – one possible approach to sharing these data is via regular webinars/research presentations; and (c) highly sensitive surveillance data, for example, known only to government officials and individuals on national suicide prevention strategy groups who have agreed not to disclose them. The latter data are unlikely to be shareable, but it will be important to consider approaches to share broad findings to give those working in different settings the opportunity to act pre-emptively and before local data are available. 

Facilities for sharing data/measures/protocols/pre-peer-reviewed manuscripts (e.g., the Open Science Framework and PsyArXiv) are possible options for building a repository of research that can have a digital object identifier (DOI) and thus are traceable and citable. *Crisis* now also publishes Registered Reports, which allow authors to submit research protocols for review before the research is conducted. 

## Conclusion 

The unique challenges posed by the current pandemic require suicide researchers to collaborate in order to understand the impact of COVID-19 on suicide and suicidal behavior and effective ways of mitigating the risk. We urge colleagues to complete the recently launched register of suicide prevention research studies to facilitate this (https://www.iasp.info/covid-19/covid-19-suicide-re​search-studies). In a challenging economic environment, suicide researchers will need to advocate strongly for the importance of the issues we have identified and make sure the research that is conducted is of the highest possible quality and ethical standard to inform public health, policy, and health-care responses. Lessons learned and subsequent changes made will contribute to improving response plans for future possible waves in this pandemic and future pandemics. The establishment of the International COVID-19 Suicide Prevention Research Collaboration is an important contribution to this effort and we ask suicide researchers particularly from regions currently not represented to join us.

## Notes

**Authorship:** TN, DG, EA, and JP contributed equally. The other authors are given in alphabetical order. International COVID-19 Suicide Prevention Research Collaboration (ICSPRC) group members: Pablo Analuisa, Louis Appleby, Ella Arensman, Jose Luis Ayuso-Mateos, Jason Bantjes, Jose Bertolote, Eric Caine, Lai Fong Chan, Shu-Sen Chang, Ying-Yeh Chen, Helen Christensen, Rakhi Dandona, Diego De Leo, Michael Eddleston, Annette Erlangsen, David Gunnell, Jill Harkavy-Friedman, Keith Hawton, Ann John, Fabrice Jollant, Nav Kapur, Murad Khan, Olivia J. Kirtley, Duleeka Knipe, Kairi Kolves, Flemming Konradsen, Shiwei Liu, Sally McManus, Lars Mehlum, Matt Miller, Ellenor Mittendorfer-Rutz, Paul Moran, Jacqui Morrissey, Christine Moutier, Thomas Niederkrotenthaler, Emma Nielsen, Merete Nordentoft, Rory O'Connor, Siobhan O'Neill, Maria Oquendo, Joseph Osafo, Andrew Page, Michael R. Phillips, Jane Pirkis, Steve Platt, Boris Polozhy, Maurizio Pompili, Ping Qin, Thilini Rajapakse, Mohsen Rezaeian, Barbara Schneider, Morton M. Silverman, Mark Sinyor, Steven Stack, Ellen Townsend, Gustavo Turecki, Michiko Ueda, Lakshmi Vijayakumar, Paul Yip, Gil Zalsman.

## References

[c1] Armstrong, S. (2020). Research on COVID-19 is suffering "imperfect incentives at every stage." *BMJ*, *369*. 10.1136/bmj.m2045 32467100

[c2] Brooks, S. K., Webster, R. K., Smith, L. E., Woodland, L., Wessely, S., Greenberg, N., & Rubin, G. J. (2020). The psychological impact of quarantine and how to reduce it: rapid review of the evidence. *Lancet*, *395*, 912–920. 10.1016/S0140-6736(20)30460-8 32112714 PMC7158942

[c3] Chang, S. S., Stuckler, S., Yip, P., & Gunnell, D. (2013). Impact of 2008 global economic crisis on suicide: Time trend study in 54 countries. *BMJ*, *347*, f5239. 10.1136/bmj.f5239 24046155 PMC3776046

[c4] Cheung, Y. T., Chau, P. H., & Yip, P. S. (2008). A revisit on older adults suicides and severe acute respiratory syndrome (SARS) epidemic in Hong Kong. *International Journal of Geriatric Psychiatry*, *23*, 1231–1238. 10.1002/gps.2056 18500689

[c5] De Leo, D., & Trabucchi, M. (2020). COVID-19 and the fears of Italian senior citizens. *International Journal of Environmental Research and Public Health*, *17*(10), 3572. 10.3390/ijerph17103572 PMC7277474 32443683

[c6] De Sousa, A., Mohandas, E., & Javed, A. (2020). Psychological interventions during COVID-19: Challenges for low and middle income countries. *Asian Journal of Psychiatry*. Advance online publication. 10.1016/j.ajp.2020.102128 PMC7195042 32380441

[c7] Deutsche Welle. (2020, June 3). *Is social distancing during coronavirus causing more suicides?* Retrieved from https://www.dw.com/en/is-social-distancing-during-coronavirus-causing-​more-suicides/a-53584282

[c8] Devitt, P. (2020). Can we expect an increased suicide rate due to COVID-19? *Irish Journal of Psychological Medicine*. Advance online publication. 10.1017/ipm.2020.46 PMC7287306 32434598

[c9] Fancourt, D., Bu, F., Mak, H. W., & Steptoe, A. (2020). COVID-19 social study. Nuffield Foundation. Retrieved from https://www.nuf​fieldfoundation.org/project/COVID-19-social-study

[c10] Gunnell, D., Appleby, L., Arensman, E., Hawton, K., John, A., & Kapur, N. (2020). Suicide risk and prevention during the COVID-19 pandemic. *The Lancet Psychiatry*. 10.1016/S2215-0366(20)30171-1 PMC7173821 32330430

[c11] Holmes, E. A., O'Connor, R. C., Perry, V. H., Tracey, I., Wessely, S., Arseneault, L., … Bullmore, E. (2020). Multidisciplinary research priorities for the COVID-19 pandemic: A call for action for mental health science. *The Lancet Psychiatry*, *7*, 547–560. 10.1016/S2215-0366(20)30168-1 32304649 PMC7159850

[c12] International Association for Suicide Prevention. (2020a). *Helping workplaces & professional associations through the COVID-10 crisis*. Retrieved from https://www.iasp.info/pdf/2020_briefing_​statement_helping_workplaces_during_COVID19.pdf

[c13] International Association for Suicide Prevention. (2020b). *Reporting on suicide during the COVID-19 pandemic*. Retrieved from https://www.iasp.info/pdf/2020_Briefing_Statement_Report​ing_on_Suicide_During_COVID19.pdf

[c14] John, A., Eyles, E., McGuinness, L. A., Okolie, C., Schmidt, L., Webb, R. T., … Higgins, J. P. T. (2020). The impact of the COVID-19 pandemic on self-harm and suicidal behaviour: Protocol for a living systematic review. [version 1; peer review: 1 approved with reservations]. *F1000Research*, *9*, 644. 10.12688/f1000research.24274.1 PMC7871358 33604025

[c15] Kawohl, W., & Nordt, C. (2020). COVID-19, unemployment, and suicide. *The Lancet Psychiatry*, *7*, 389–390. 32353269 10.1016/S2215-0366(20)30141-3 PMC7185950

[c16] Knipe, D., Evans, H., Marchant, A., Gunnell, D., & John, A. (2020). Mapping population mental health concerns related to COVID-19 and the consequences of physical distancing: A Google trends analysis [version 2; peer review: 2 approved]. *Wellcome Open Research*, *5*, 82. 10.12688/wellcomeopenres.15870.2 32671230 PMC7331103

[c17] Liu, Q., Luo, D., Haase, J. E., Guo, Q., Wang, X. Q., Liu, S., … Yang, B. X. (2020). The experiences of health-care providers during the COVID-19 crisis in China: A qualitative study. *The Lancet Global Health*, *8*(6), e790–e798. 10.1016/S2214-109X(20)30204-7 32573443 PMC7190296

[c18] Mamun, M. A., & Ullah, I. (2020). COVID-19 suicides in Pakistan, dying off not COVID-19 fear but poverty? The forthcoming economic challenges for a developing country. *Brain, Behavior, and Immunity*, *87*, 163–166. 10.1016/j.bbi.2020.05.028 PMC7212955 32407859

[c19] Mars, B., Heron, J., Klonsky, E. D., Klonsky, D., Moran, P., O'Connor, R., … Gunnell, D. (2019). Predictors of future suicide attempt among adolescents with suicidal thoughts or non-suicidal self-harm: A population-based birth cohort study. *The Lancet Psychiatry*, *6*, 327–337. 10.1016/S2215-0366(19)30030-6 30879972 PMC6494973

[c20] May, A. M., & Klonsky, E. D. (2016). What distinguishes suicide attempters from suicide ideators? A meta–analysis of potential factors. *Clinical Psychology*, *23*(1), 5–20. 10.1111/cpsp.12136

[c21] McIntyre, R. S., & Lee, Y. (2020). Preventing suicide in the context of the COVID-19 pandemic. *World Psychiatry*, *19*, 250-251. 10.1002/wps.20767 32394579 PMC7214950

[c22] Moser, D. A., Glaus, J., Frangou, S., & Schechter, D. S. (2020). Years of life lost due to the psychosocial consequences of COVID19 mitigation strategies based on Swiss data. *European Psychiatry*, *63*(1), e58. 10.1192/j.eurpsy.2020.56 32466820 PMC7303469

[c23] New Zealand Herald. (2020, May 19). *COVID 19 coronavirus: Fewer suicides during lockdown level-4*. Retrieved from https://www.nzherald.co.nz/nz/news/article.cfm?c_id=1&objectid​=12333030

[c24] Norström, T., & Grönqvist, H. (2015). The Great Recession, unemployment and suicide. *Journal of Epidemiology and Community Health*, *69*, 110–116. 10.1136/jech-2014-204602 25339416 PMC4316842

[c25] O'Connor, R. C., Hotopf, M., Worthman, C. M., Perry, V. H., Tracey, I., Wessely, S., … Holmes, E. A. (2020). Multidisciplinary research priorities for the COVID-19 pandemic: authors' reply. *Lancet Psychiatry*, *7*, e44-e45. 10.1016/S2215-0366(20)30247-9 32563319 PMC7302786

[c26] Pierce, M., McManus, S., Jessop, C., John, A., Hotopf, M., Ford, T., … Abel, K. M. (2020). Says who? The significance of sampling in mental health surveys during COVID-19. *The Lancet Psychiatry*, *7*(7), 567. 10.1016/S2215-0366(20)30237-6 32502467 PMC7266586

[c27] Reger, M. A., Stanley, I. H., & Joiner, T. W. (2020). Suicide mortality and coronavirus disease 2019 – a perfect storm? *JAMA Psychiatry*. Advance online publication. 10.1001/jamapsychiatry.2020.1060 32275300

[c28] Rogers, J. P., Chesney, E., Oliver, D., Pollak, T. A., McGuire, P., Fusar-Poli, P., … David, A. S. (2020). Psychiatric and neuropsychiatric presentations associated with severe coronavirus infections: A systematic review and meta-analysis with comparison to the COVID-19 pandemic. *The Lancet Psychiatry*, *7*(7), 611–627. 10.1016/S2215-0366(20)30203-0 32437679 PMC7234781

[c29] Royal College of Psychiatrists. (2020). *Psychiatrists see alarming rise in patients needing urgent and emergency care and forecast a 'tsunami' of mental illness*. Retrieved from https://www.rcpsych.ac.uk/news-and-features/latest-news/detail/​2020/05/15/psychiatrists-see-alarming-rise-in-patients-needing-urgent-and-emergency-care

[c30] Sinyor, M., Spittal, M. J., & Niederkrotenthaler, T. (2020). Changes in suicide and resilience-related Google searches during the early stages of the COVID-19 pandemic. *Canadian Journal of Psychiatry*. Advance online publication. 10.1177/0706743720933426 PMC7502879 32524848

[c31] The Guardian. (2020, May 14). *Japan suicides decline as COVID-19 lockdown causes shift in stress factors*. Retrieved from https://www.theguardian.com/world/2020/may/​14/japan-suicides-fall-sharply-as-COVID-19-lockdown-causes-​shift-in-stress-factors

[c32] Torjesen, I. (2020). COVID-19: Doctors need proper mental health support, says BMA. *BMJ*, *369*, m2192. 10.1136/bmj.m2192 32482682

[c33] Townsend, E., Nielsen, E., Allister, R., & Cassidy, S. A. (2020). Key ethical questions for research during the COVID-19 pandemic. *The Lancet Psychiatry*, *7*(5), 381–383. 10.1016/S2215-0366(20)30150-4 32353264 PMC7185919

[c34] Tran, U. S., Andel, R., Niederkrotenthaler, T., Till, B., Ajdacic-Gross, V., & Voracek, M. (2017). Low validity of Google Trends for behavioral forecasting of national suicide rates. *PLoSONE*, *12*(8), e0183149. 10.1371/journal.pone.0183149 PMC5558943 28813490

[c35] United Nations. (2020a). *Policy brief: COVID-19 and the need for action on mental health*. Retrieved from https://www.un.org/sites/un2.un.org/files/un_policy_brief-COVID_and_mental_health_final.pdf

[c36] United Nations. (2020b). *UN chief calls for 'solidarity, unity and hope' in battling COVID-19 pandemic*. Retrieved from https://news.un.org/en/story/2020/04/1062972

[c37] Van Bavel, J. J., Baicker, K., Boggio, P. S., Capraro, V., Cichocka, A., Cikara, M., … Willer, R. (2020). Using social and behavioral science to support COVID-19 pandemic response. *Nature Human Behavior*, *4*, 460–471. 10.1038/s41562-020-0884-z 32355299

[c38] Wand, A. P. F., Zhong, B. L., Chiu, H. F. K., Draper, B., & De Leo, D. (2020). COVID-19: The implications for suicide in older adults. *International Psychogeriatrics*. 10.1017/S1041610220000770 PMC7235297 32349837

[c39] Wasserman, I. M. (1992). The impact of epidemic, war, prohibition and media on suicide: United States, 1910–1920. *Suicide and Life-Threatening Behavior*, *22*, 240–254. 10.1111/j.1943-278X.1992.tb00231.x 1626335

[c40] Wu, Y., Xu, X., Chen, Z., Duan, J., Hashimoto, K., Yang, L., … Yang, C. (2020). Nervous system involvement after infection with COVID-19 and other coronaviruses. *Brain, Behavior, and Immunity*, *87*, 18–22. 10.1016/j.bbi.2020.03.031 PMC7146689 32240762

[c41] World Health Organization. (2014). *Preventing suicide: A global imperative*. Geneva, Switzerland: Author. Retrieved from https://www.who.int/mental_health/suicide-prevention/world_report_​2014/en/

[c42] World Health Organization. (2016). *Practice manual for establishing and maintaining surveillance systems for suicide attempts and self-harm*. Retrieved from https://www.who.int/mental_health/suicide-prevention/attempts_surveillance_systems/en/

[c43] Worldometers. (2020). *COVID-19 Coronavirus pandemic*. Retrieved from https://www.worldometers.info/coronavirus/

[c44] Yao, H., Chen, J. H., & Xu, Y. F. (2020). Patients with mental health disorders in the COVID-19 epidemic. *The Lancet Psychiatry*, *7*(4), e21. 10.1016/S2215-0366(20)30090-0 32199510 PMC7269717

[c45] Yip, P. S. F., & Chau, P. H. (2020). Physical distancing and emotional closeness amidst COVID-19. *Crisis*. Advance online publication. 10.1027/0227-5910/a000710 32299225

[c46] Zhang, C., Shi, L., & Wang, F. S. (2020). Liver injury in COVID-19: Management and challenges. *The Lancet Gastroenterology and Hepatology*, *5*(5), 428–430. 10.1016/S2468-1253(20)30057-1 32145190 PMC7129165

[c47] Zortea, T. C., Brenna, C. T. A., Joyce, M., McClelland, H., Tippett, M., Tran, M., … Platt, S. (2020). *The impact of infectious disease-​related public health emergencies on suicide, suicidal behavior, and suicidal thoughts: A systematic review*. Retrieved from https://www.crd.york.ac.uk/PROSPERO/display_record.php?​RecordID=187013 10.1027/0227-5910/a000753 PMC8689932 33063542

